# A case report of Gitelman syndrome in children

**DOI:** 10.1097/MD.0000000000033509

**Published:** 2023-04-14

**Authors:** Jing Ying, Haixia Wu, Ruizhong Zhang, Pengmei Wu, Fengxuan Sui, Zilong Li

**Affiliations:** a Department of Pediatrics, Affiliated Shenzhen Longhua People’s Hospital, Southern Medical University, Longhua, Shenzhen, Guangdong, P. R. China.

**Keywords:** children, gene sequencing, giltelman syndrome, hypokalemia, hypomagnesemia

## Abstract

Giltelman syndrome (GS) is an autosomal recessive infectious disease, which is caused by the mutation of SLC12A3 gene encoding thiazide diuretic sensitive sodium chloride cotransporter located in the distal convoluted tubule of the kidney.

**Patient concerns:**

A 7-year-old and 3-month-old male patient has poor appetite, slow growth in height and body weight since the age of 3, body weight: 16 kg (−3 standard deviation), height: 110 cm (−3 standard deviation), normal exercise ability and intelligence. One year ago, he was diagnosed with hypokalemia. After potassium supplement treatment, the blood potassium returned to normal. The patient developed abdominal pain, vomiting, limb weakness, and tetany 1 day before admission.

**Diagnoses:**

After admission examination, the patient was found to have hypokalemia (2.27–2.88 mmol/L), hypomagnesemia (0.47 mmol/L), hypophosphatemia (1.17 mmol/L), hypocalcemia (1.06 mmol/24 hours), and metabolic alkalosis (PH 7.60). The blood pressure is normal, and the concentration of aldosterone is 791.63 pg/mL. The adrenocorticotropic hormone and cortisol detected at 8 am are 4.95 pmol/L and 275.09 nmol/L, respectively. Twenty-four hours of urine potassium is 32.52 mmol. Gene sequencing results showed 2 pathogenic variants in the GS-related SLC12A3 gene, which are related to the phenotype of the subject.

**Interventions:**

After admission, the patients were given potassium and magnesium supplements, as well as oral spironolactone. The symptoms of limb weakness and tetany were significantly relieved. After discharge, the patients continued to maintain treatment to keep the blood potassium at more than 3.0 mmol/L, and the blood magnesium at more than 0.6 mmol/L.

**Outcomes:**

Follow-up at 1 month after discharge, in the patient’s self-description, he had no symptoms such as limb weakness and tetany, and his height was increased by 1 cm and the body weight increased by 1.5 kg.

**Lessons:**

For patients with hypokalemia, hypomagnesemia, and metabolic alkalosis, the possibility of GS should be given priority. After the diagnosed by gene sequencing of SLC12A3 gene, potassium and magnesium supplementation could significantly improve symptoms.

## 1. Introduction

Giltelman syndrome is a rare hereditary renal tubular disease. Patients may have clinical symptoms such as hypokalemia, hypomagnesemia, metabolic alkalosis, and hypocalcemia. In addition, due to long-term electrolyte disorder and acid-base imbalance, it will lead to growth retardation and kidney damage in children.^[1]^ The pathogenesis of giltelman syndrome (GS) is due to the mutation of the gene SLC12A3 encoding the sodium chloride cotransporter sensitive to thiazide diuretics localized in the distal convoluted tubules of the kidney. GS is a rare disease worldwide, and the incidence rate is 1 to 10/40,000. The incidence rate of Asian population is higher than that of other regions.^[1]^ Most patients with GS have a good prognosis after effective treatment, but there are still serious cases reported. Here we report a case of GS diagnosed and treated in the Department of Pediatrics, Affiliated Shenzhen Longhua People’s Hospital, Southern Medical University.

## 2. Case description

A 7-year-old and 3-month-old male patient was admitted to the pediatric ward due to abdominal pain, vomiting, weakness of limbs, and tetany for 1 day. The patient suffered from abdominal pain and repeated vomiting 1 day before, followed by weakness of limbs and tetany. The patient was in poor mental state, without obvious dehydration appearance, and was admitted to the emergency hospital with blood potassium of 2.27 mmol/L. At admission: body temperature 36.7°C, heart rate 122 times/minutes, breathing 22 times/minutes, blood pressure 98/59 mm Hg, height 110 cm (−3 standard deviation), weight 16 kg (−3 standard deviation), normal exercise ability and intelligence. No abnormalities found in thyroid palpation, lung auscultation, heart sound, and rhythm during the physical examination. Limb muscle strength is grade 4. According to the guardian’s description, the patient has poor appetite, slow growth in height and weight since the age of 3. One year ago, the patient was diagnosed with hypokalemia. After potassium supplement treatment, the blood potassium returned to normal. No history of tumor and metabolic disease. No medication (such as diuretics) has been taken recently. No similar medical history among the family members.

**Figure 1.**
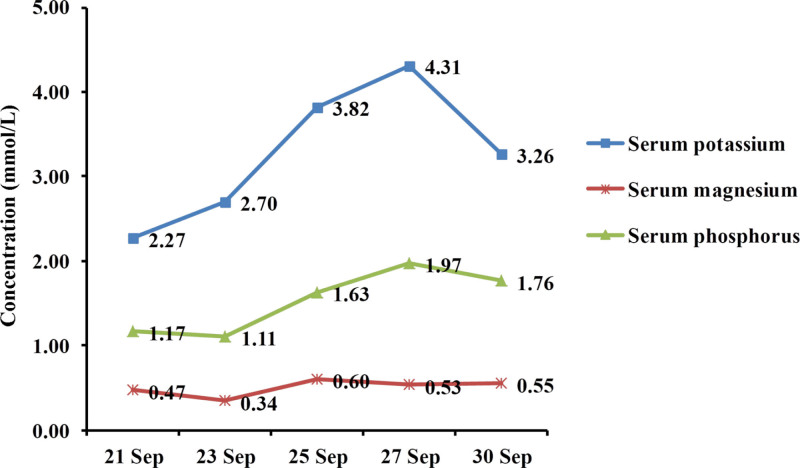
Laboratory examination of Serum electrolyte. Blue line for serum potassium, brown line for serum magnesium, and green line for serum phosphorus.

**Figure 2.**
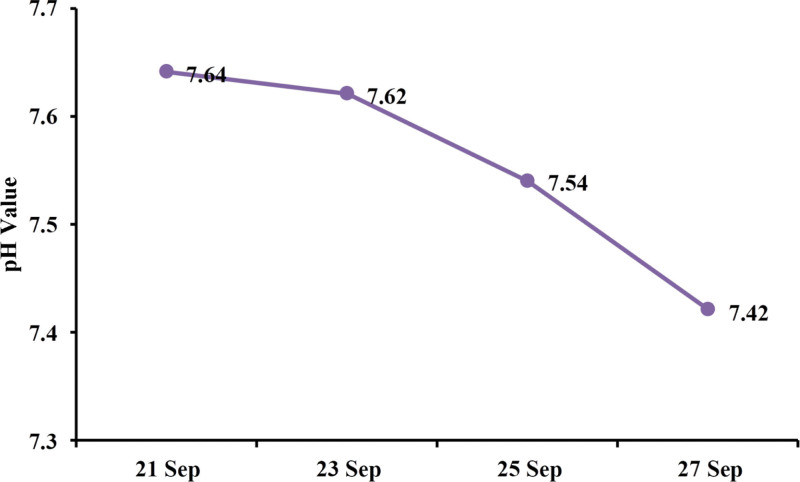
pH value.

After admission, the doctor in charge supplemented some laboratory examination, and the results showed that the patient’s blood potassium was 2.70 mmol/L, blood magnesium 0.34 mmol/L, blood phosphorus 1.11 mmol/L (Fig. 1, September 23, 2022), and PH 7.62 (Fig. 2, September 23, 2022). Subsequently, the patient was immediately given intravenous potassium and magnesium supplement, and improvement of acid-base imbalance.

The clinical symptoms of the patient were relieved, but the blood potassium was maintained at 2.27 to 2.70 mmol/L, the blood magnesium at 0.34 to 0.47 mmol/L (Fig. 1), and the PH value at 7.62 to 7.64 (Fig. 2). The electrocardiogram showed that the sinus tachycardia, and the T wave of I, II, aVL, aVF, V3 to V6 were low and flat.

After case discussion in the department, we further improved some laboratory examination. The results showed that the levels of aldosterone and renin are increased. However, the cortisol and adrenocortical hormone are normal. No abnormality was found in liver function, kidney function, myocardial enzyme, and thyroid function. The 24-hour urine electrolyte analysis showed that urinary potassium increased (32.52 mmol/24 hours) and urinary calcium decreased (1.06 mmol/24 hours) (Table 1).

**Table 1:** Laboratory examination.

	Value	Normal range
pH (Urine)	7.5	5.0–8.0
Urine specific gravity	1.011	1.005–1.030
Urinary protein	Negative	Negative
Urea nitrogen, mmol/L	2.52	1.8–6.4
Creatinine, mmol/L	2.52	17.7–61.9
Blood sugar, mmol/L	5.4	3.6–5.8
24-hour urine potassium, mmol/d	32.52	
24-hour urine calcium, mmol/d	1.06	
Parathyroid hormone, pg/mL	11.80	15.00–88.0
Adrenocorticotropic hormone, pmol/L	4.95	1.60–13.90
Cortisol (8 am), nmol/L	275.09	185–624
Aldosterone (orthostatic), pg/mL	791	40–310
Aldosterone (clinostatism), pg/mL	342.56	40–310
Renin	25.50	

After obtaining informed consent, blood samples were sent out to BGI (Shenzhen, China) for whole exon sequencing of patients and relatives. As shown in Table 2, 2 variants (c.1331delA and c.975_976insT) were detected on the GS-related SLC12A3 gene, which are related to the phenotype of the subject.

**Table 2:** Gene sequencing.

Gene	Chromosome position	Transcript no nucleotide change (amino acid change)	Gene subregion	Genotype	ACMG variation rating	Sequencing results of relatives	
						Father	Mother
SLC12A3	chr16:56,913,135	NM 001126108.1 c.1331delA (p.Ty444Serfs*4)	EX10/CDS10	Heterozygote	Suspected disease	Heterozygote	Not detected
SLC12A3	Chr16:56906,573–56,906,574	NM 001126108.1 c.975 976insT (p.Val326Cysfs*7)	EX8/CDS8	Heterozygote	Suspected disease	Not detected	Not detected

According to the clinical manifestation and gene sequencing results of the patient, the diagnose of the patient is Gitelman syndrome. Therefore, the patients were given potassium and magnesium supplements, as well as oral spironolactone to reduce the excretion of potassium in urine. After treatment, the symptoms of limb weakness and tetany basically disappeared, and the serum potassium, serum magnesium, serum phosphorus and pH were also basically stable.

After discharge, we followed up the patient and adjusted the drug dosage to keep the blood potassium the blood magnesium remained above 3.0 mmol/L and 0.6 mmol/L, respectively. Moreover, with the stability of serum electrolyte level and renal function, the problem of growth retardation has also been improved. One month after discharge, the patient’s height increased by 1 cm and body weight increased by 1.5kg. After 2 months, the patient’s height increased by 4 cm and body weight increased by 2.5 kg. In addition, the patient’s psychological and emotional state remained stable during the medication, no other complications and side effects of drugs was founded. Therefore, we concluded that the treatment effect and prognosis of the patient is good, and we will continue to follow up and monitor the patient regularly in the future.

## 3. Discussion

The main clinical symptoms of the patient are abdominal pain, vomiting, limb weakness, and tetany. After supplemented the routine electrolyte examination, we found that the most prominent problem was abnormal hypokalemia. The cause of hypokalemia should take into account insufficient potassium intake, increased potassium loss, and abnormal potassium distribution. The patient has poor appetite, which may lead to insufficient potassium intake, but this is not enough to explain the severe hypokalemia. It is necessary to further find out whether there is the cause of excessive loss, which includes gastrointestinal loss, kidney loss, and skin loss. Although the patient has symptoms of acute gastroenteritis, but this could not explain the clinical manifestations of severe hypokalemia, hypomagnesium, and metabolic alkalosis.

After case discussion in the department, we tried to determine the patient’s 24-hour urine potassium to check kidney potassium loss. On the other hand, the patient no history of medication of potassium excretion diuretics and large doses of insulin, no hyperthyroidism, no diabetes ketoacidosis, no medication of β2 receptor agonists, which can lead to potassium ion intracellular migration. According to the clinical characteristics with kidney potassium loss, metabolic alkalosis, and normal blood pressure, the diagnose of primary aldosteronism, renal tubular acidosis, renin-secreting tumor, renal artery stenosis, and other diseases can be ruled out. The etiology of the patient ultimately points to GS.

Gene sequencing of the whole exon for the patient and his family members showed that 2 variants (c.1331delA and c.975_976insT) were detected on the GS-related SLC12A3 gene, which constituted a complex heterozygous mutation, may be related to the subject’s phenotype. We reviewed the relevant literature and gene libraries, but neither of the 2 variant phenotypes was included in the genome database of GS. According to the ACMG guidelines,^[2]^ the mutations were determined to be suspected pathogenic variant. However, the clinical manifestation and treatment effect of the patient were closely consistent with the diagnosis of GS. Therefore, this is a new type of variation. It is worth mentioned that, according to the gene sequencing results of his paraents, 1 heterozygous variation (c.1331delA) came from the father, while the other heterozygous variation (c.975_976insT) could not be found in the parents genes. This may be due to the patient’s own genetic variation, and the 2 variations formed a complex heterozygous mutation, which eventually led to the disease. However, single heterozygous mutations were also reported in some patients,^[3,4]^ which can be further analyzed for intron mutations, large segment deletion, and duplication of genes. In addition, it is reported that the pathogenesis of GS may be related to the mutations of CLCNKB gene^[5,6]^ and HNF1B gene,^[7]^ but we did not conduct the genes sequencing since we did not get the consent of the patient’s guardians.

GS is a rare autosomal recessive renal tubular disease. Since it was first reported in 1966,^[8]^ with the continuous research of many scholars, our understanding of GS has gradually deepened. The pathogenesis of GS is due to the mutation of the gene SLC12A3 encoding the sodium chloride cotransporter sensitive to thiazide diuretics localized in the distal convoluted tubules of the kidney, resulting in the malabsorption of Na^+^ and Cl^−^ in the distal convoluted tubules. The decrease of reabsorption of sodium chloride can further activate the renin-angiotensin-aldosterone system, while the increase of aldosterone can increase the reabsorption of sodium ions and water molecules, but also cause the decrease of potassium ion absorption, leading to hypokalemia and metabolic alkalosis. In addition, the increase of aldosterone secretion will also lead to the decrease of intestinal magnesium absorption and renal tubular magnesium ion reabsorption, which will increase urinary magnesium and lead to hypomagnesemia. Therefore, the main clinical manifestations of GS patients are hypokalemia, hypomagnesemia, hypocalcemia, metabolic alkalosis, myasthenia, tetany, and children also have growth retardation. Some of them patients also have proteinuria and renal function damage.^[1]^

The treatment of GS is aimed to improve the symptoms and quality of life of patients. The mainly treatment method is life-long electrolyte replacement therapy. In addition to dietary supplements in daily life, it depends on potassium and magnesium supplements to maintain the stability of serum potassium and magnesium. It is generally believed that the serum potassium in GS patients should be more than 3.0 mmol/L, and the serum magnesium should be more than 0.6 mmol/L.^[1]^ When the patient has stubborn electrolyte disorder, or needs to be treated with high dose and/or intravenous potassium and magnesium supplements, potassium sparing diuretics, COX inhibitors, ACEI/ARB, and other drugs can be used together to maintain electrolyte stability and reduce the dosage of substitute drugs.^[9,10]^

In conclusion, as a rare disease, GS is easy to be misdiagnosed or missed due to lack of understanding. For patients with hypokalemia, hypomagnesemia, and metabolic alkalosis that are difficult to relieved, the possibility of GS should be considered. It is necessary to conduct gene sequencing to clarify the cause of disease and carry out electrolyte replacement therapy to improve the clinical symptoms and quality of life of patients.

## Author contributions

**Conceptualization:** Jing Ying.

**Data curation:** Haixia Wu.

**Investigation:** Jing Ying.

**Methodology:** Ruizhong Zhang.

**Resources:** Pengmei Wu.

**Supervision:** Fengxuan Sui.

**Writing – original draft:** Zilong Li.

**Writing – review & editing:** Zilong Li.
